# Factors associated with social functioning by relapse frequency in Japanese outpatients with schizophrenia: the Multicenter Treatment Survey and Assessments for Schizophrenia in Psychiatric Clinics (MUSASI)

**DOI:** 10.1017/S0033291726104231

**Published:** 2026-05-29

**Authors:** Yoshiteru Takekita, Eiichi Katsumoto, Naoto Adachi, Yukihisa Kubota, Koji Edagawa, Takaharu Azekawa, Hitoshi Ueda, Tatsuji Tamura, Seiji Hongo, Eiichiro Goto, Hirohisa Hida, Kazuhira Miki, Chiyo Fujii, Koichiro Watanabe, Masaki Kato, Norio Yasui-Furukori, Hiroyoshi Takeuchi

**Affiliations:** 1 Department of Neuropsychiatry, Faculty of Medicine, Kansai Medical University, Hirakata, Japan; 2 Katsumoto Mental Clinic, Osaka, Japan; 3 Adachi Mental Clinic, Sapporo, Japan; 4 Chuokoen Clinic, Fuji, Japan; 5 Edagawa Clinic, Tokushima, Japan; 6 Shioiri Mental Clinic, Yokosuka, Japan; 7 Morioka Cocoro Clinic, Morioka, Japan; 8 Tamura Mental Clinic, Hiroshima, Japan; 9 Nanko Institute of Psychiatry, Shirakawa, Japan; 10 Shinwado Goto Clinic, Fukuoka, Japan; 11 Hida Clinic, Nagareyama, Japan; 12 Miki Mental Clinic, Yokohama, Japan; 13 National Center of Neurology and Psychiatry, Kodaira, Japan; 14 Department of Neuropsychiatry, Kyorin University School of Medicine, Mitaka, Japan; 15 Department of Psychiatry, Dokkyo Medical University School of Medicine, Mibu, Japan; 16 Department of Psychiatry, Chiba University Graduate School of Medicine, Chiba, Japan

**Keywords:** outpatients, recurrence, relapse, risk factors, schizophrenia, social functioning

## Abstract

**Background:**

Impaired social functioning substantially affects the quality of life of patients with schizophrenia; however, it remains unclear whether the factors associated with social functioning differ according to relapse frequency. This study aimed to examine the differences in these factors among Japanese outpatients with schizophrenia, stratified by relapse frequency.

**Methods:**

This nationwide cross-sectional study, the Multicenter Treatment Survey and Assessments for Schizophrenia in Psychiatric Clinics (MUSASI), was conducted in 330 psychiatric clinics in Japan between September and October 2023. A total of 10,081 patients diagnosed with schizophrenia-related disorders were analyzed. Patients were categorized as nonrelapsers, low-frequency relapsers (1–2 relapses), or high-frequency relapsers (≥3 relapses). Social functioning was assessed using the Social and Occupational Functioning Assessment Scale, with scores ≥61 defined as high functioning.

**Results:**

This study included 3,670 nonrelapsers, 4,428 low-frequency relapsers, and 1,983 high-frequency relapsers. Overall, 55.8% (*n* = 5,631) of patients were classified as having high social functioning. Across all groups, employment, shorter periods of instability during the past year, lower Clinical Global Impression–Severity scores, and fewer negative symptoms were significantly associated with higher social functioning. Group-specific associations were also observed: in nonrelapsers and low-frequency relapsers, positive symptoms and medication-related factors were relevant, whereas in low- and high-frequency relapsers, marital history was associated, and in high-frequency relapsers, the absence of tardive dyskinesia emerged as a factor.

**Conclusions:**

Factors associated with social functioning differed according to relapse frequency, highlighting the need for relapse frequency-based, stratified intervention strategies.

## Introduction

Schizophrenia is one of the most severe psychiatric disorders. It presents with diverse problems, including positive symptoms such as hallucinations and delusions, negative symptoms such as affective flattening and loss of motivation, and cognitive impairments such as deficits in attention, memory, and executive function, and follows a chronic course (Jauhar, Johnstone, & McKenna, 2022). Additionally, social functioning is reportedly impaired in 65–82% of patients (Holt-Lunstad et al., 2015; Pjescic et al., 2014; Velthorst et al., 2016, 2017). Social functioning includes a wide range of domains such as interpersonal relationships, social roles, regular and independent living, and participation in leisure and community activities (Dziwota, Stepulak, Wloszczak-Szubzda, & Olajossy, 2018; Hoier et al., 2024). Improvement in social functioning is an important clinical indicator that determines patients’ quality of life (QoL) and recovery (Trompenaars et al., 2007). Together with improvement in symptoms and cognition, it has become one of the most important outcomes of recent schizophrenia treatment. Therefore, identifying and understanding the factors that affect social functioning are crucial for patient support.

Previous studies have identified multiple factors associated with social functioning, including negative symptoms, cognitive impairment, insight, medication adherence, and social support, which may interact and influence social adaptation (de Winter et al., 2025; Erol, Delibas, Bora, & Mete, 2015; Mohamed et al., 2009; Norman et al., 2012). In contrast, schizophrenia is associated with a high relapse rate, and more than half of patients experience relapse during the course of illness (Carbon & Correll, 2014). Relapses have detrimental effects on cognitive function and have severe psychosocial consequences (Emsley, Chiliza, & Asmal, 2013; Hori, Atake, Katsuki, & Yoshimura, 2021). Thus, in patients with frequent relapses, the factors affecting social functioning may differ from those in patients with few or no relapses. However, little is known about how factors influencing social functioning differ according to relapse status. This knowledge gap presents a challenge in planning individualized support and developing treatment strategies. Therefore, this study aimed to investigate whether factors related to social functioning differ depending on the relapse frequency in Japanese outpatients with schizophrenia.

## Methods

### Study design and participants

The Multicenter Treatment Survey and Assessments for Schizophrenia in Psychiatric Clinics (MUSASI) was a cross-sectional study conducted from September to October 2023 in psychiatric clinics across Japan. Participants were patients diagnosed with schizophrenia, schizoaffective disorder, or delusional disorder (ICD-10, F2), who were receiving treatment at the clinics. Most psychiatrists who participated in this study were certified by the Japanese Society of Psychiatry and Neurology and/or were designated psychiatrists approved by the Ministry of Health, Labour, and Welfare of Japan. This cross-sectional study was reported in accordance with the STROBE guidelines; the completed STROBE checklist is provided in Supplementary Table S1.

### Study procedures

Psychiatrists were asked to retrospectively review the medical records of consecutive patients with an F2 diagnosis who visited the clinic during the study period, beginning with the first patient seen and completing the questionnaire. The questionnaire included questions on demographic and clinical characteristics (sex, age, employment status, public assistance, cohabitation, educational attainment, height, weight, and marital history), diagnosis, age at onset, stability of visits and medication, unstable periods during the past year, psychiatric hospitalization during the past year, relapse frequency, Clinical Global Impression–Severity (CGI-S) (Guy, 1976), modified Brief Evaluation of Psychosis Symptom Domains (BE-PSD) (Takeuchi et al., 2016), modified Social and Occupational Functioning Assessment Scale (SOFAS) (Goldman, Skodol, & Lave, 1992), pharmacological treatment, and side effects.

Relapse frequency was categorized as follows: nonrelapsers (no relapse), low-frequency relapsers (1–2 relapses), and high-frequency relapsers (≥3 relapses). The modified BE-PSD, similar to the original BE-PSD, assessed five symptom domains (psychotic symptoms, disorganized thinking, negative symptoms, excitement/mania, and depression/anxiety) on a five-point scale (0 = none, 1 = mild, 2 = moderate, 3 = severe, and 4 = very severe). The BE-PSD has been shown to correlate well with the Positive and Negative Syndrome Scale (PANSS) and the Clinical Global Impression-Severity (CGI-S) (Takeuchi et al., in press). The modified SOFAS rated functioning in four categories: 0:1–40, 1:41–60, 2:61–80, and 3:81–100.

### Statistical analyses

Following the classification of functional remission proposed by Schennach-Wolff et al., a SOFAS cutoff score of 61 was adopted to divide patients into “high social functioning” and “low social functioning” groups (Schennach-Wolff et al., 2009). A score of 61 represents a clinically meaningful threshold at which patients can maintain independent living and fulfill their social roles. This cutoff allowed for a clear comparison and interpretation across relapse frequency groups.

To compare demographic and clinical characteristics among groups based on relapse frequency, Kruskal–Wallis and Pearson’s chi-square tests were used. As multiple comparisons were performed among the three groups, Bonferroni correction was applied, and *p*-values <0.0167 were considered significant.

Univariate logistic regression analyses were conducted for all patients to identify the demographic and clinical factors associated with high social functioning. Variables with *p* < 0.001 were subsequently included in multivariate logistic regression analyses and stratified according to relapse frequency (nonrelapsers, low-frequency relapsers, and high-frequency relapsers). Factors with *p* < 0.05 in the multivariate analysis were considered significantly associated with social functioning.

Unless otherwise specified, all statistical tests were two-tailed with a significance level of 0.05. All analyses were performed using SPSS version 25 (IBM Corp., Armonk, NY).

### Ethics statement

This study was conducted in accordance with the Declaration of Helsinki and the Ethical Guidelines for Medical and Biological Research Involving Human Subjects established by the Ministry of Health, Labour, and Welfare of Japan. The study protocol was reviewed and approved by the Ethics Committee of the Japanese Association of Neuro-Psychiatric Clinics (ID: 2023-4). As this was a retrospective review of medical records, the requirement for informed consent was waived; however, patients were informed and allowed to opt out of the study, which was implemented at each participating clinic through institutional bulletin board postings or clinic websites. To protect patient confidentiality, each participant was assigned a study-specific anonymized identification number to ensure that no individual could be identified.

## Results

### Demographic characteristics

Of the 1,544 facilities invited to participate, 330 clinics (21.4%) responded. Data were obtained from 10,127 patients; however, 46 patients with missing data on psychiatric symptoms or pharmacotherapy were excluded, leaving 10,081 patients for the analysis. Demographic characteristics of participants are summarized in Table 1. The mean age was 51.5 ± 13.5 years, and 46.3% (*n* = 4,664) were men. The mean age at onset was 27.3 ± 10.8 years, and the mean duration of illness was 24.2 ± 12.5 years. Regarding employment, 47.6% (*n* = 4,800) held some form of a social role. A total of 71.3% (*n* = 7,194) lived with others, and 34.9% (*n* = 3,519) had been married. The mean body mass index (BMI) was 24.2 ± 4.5 kg/m^2^. The proportion of patients with high social functioning was 55.8% (*n* = 5,631).

**Table 1.** Demographic characteristics of the study participants

	Total (*n* = 10,081)	Nonrelapsers (*n* = 3,670)	Low-frequency relapsers (*n* = 4,428)	High-frequency relapsers (*n* = 1,983)	*p*-value
Male sex [*n*, (%)]^a^	4,664 (46.3)	1,663 (45.3)	2,094 (47.3)	907 (45.7)	0.188
Mean age [years, mean (SD)]^b^	51.5 (13.5)	49.1 (14.4)	52.4 (12.8)	54.1 (12.5)	<0.001
Body weight [kg, mean (SD)]^b^	64.1 (13.7)	63.1 (13.7)	64.4 (13.4)	65.7 (14.3)	<0.001
Body mass index [mean (SD)]^b^	24.2 (4.5)	23.8 (4.5)	24.3 (4.4)	24.9 (4.7)	<0.001
Age of onset [years, mean (SD)]^b^	27.3 (10.8)	28.9 (12.5)	26.8 (9.9)	25.6 (8.8)	<0.001
Duration of illness [years, mean (SD)]^b^	24.2 (12.5)	20.1 (12.4)	25.6 (11.9)	28.5 (11.7)	<0.001
SOFAS 61–100 [*n*, (%)]^a^	5,631 (55.8)	2,205 (60.1)	2,550 (57.6)	876 (44.2)	<0.001
Severity of psychiatric symptoms [mean (SD)]^b^					
Psychotic symptoms	1.1 (1.0)	1.0 (1.0)	1.1 (0.9)	1.4 (1.0)	<0.001
Disorganized thinking	0.9 (0.9)	0.8 (0.9)	1.0 (0.87)	1.3 (1.0)	<0.001
Negative symptoms	1.3 (0.9)	1.1 (0.9)	1.3 (0.8)	1.5 (0.9)	<0.001
Excitement/mania	0.4 (0.7)	0.3 (0.6)	0.4 (0.7)	0.6 (0.8)	<0.001
Depression/anxiety	0.8 (0.8)	0.8 (0.8)	0.8 (0.8)	1.0 (0.8)	<0.001
Total antipsychotic dose [chlorpromazine equivalent, mg, mean (SD)]^b^	513.0 (462.0)	445.3 (413.7)	525.7 (476.0)	609.8 (494.3)	<0.001
Diagnosis [*n*, (%)]^a^					<0.001
Schizophrenia	8,659 (85.9)	3,154 (85.9)	3,846 (86.9)	1,659 (83.7)	
Schizotypal disorder	134 (1.3)	62 (1.7)	46 (1.0)	26 (1.3)	
Delusional disorders	516 (5.1)	258 (7.0)	202 (4.6)	56 (2.8)	
Acute and transient psychotic disorders	89 (0.9)	55 (1.5)	26 (0.6)	8 (0.4)	
Schizoaffective disorders	641 (6.4)	125 (3.4)	288 (6.5)	228 (11.5)	
Others	24 (0.2)	8 (0.2)	11 (0.2)	5 (0.3)	
Employment [*n*, (%)]^a^	4,800 (47.6)	1,755 (47.8)	2,171 (49.0)	874 (44.1)	<0.001
Public assistance [*n*, (%)]^a^	8,658 (85.9)	2,935 (80.0)	3,906 (88.2)	1,817 (91.6)	<0.001
No independent living [*n*, (%)]^a^	7,194 (71.3)	2,783 (75.8)	3,089 (69.8)	1,322 (66.7)	<0.001
Married [*n*, (%)]^a^	3,519 (34.9)	1,221 (33.3)	1,543 (34.8)	755 (39.2)	0.001
Psychotropic agents [*n*, (%)]^a^					
Long-acting injectable antipsychotics	1,209 (12.0)	279 (7.6)	585 (13.2)	345 (17.4)	<0.001
Monotherapy with antipsychotics	6,059 (60.1)	2,354 (64.1)	2,649 (59.8)	1,056 (53.3)	<0.001
Antidepressants	1,597 (15.8)	595 (16.2)	701 (15.8)	301 (15.2)	0.597
Anxiolytics/hypnotics	6,384 (63.3)	2,190 (59.7)	2,830 (63.9)	1,364 (68.8)	<0.001
Mood stabilizers	1,447 (14.4)	345 (9.4)	669 (15.1)	433 (21.8)	<0.001
Antiparkinson agent	2,820 (28.0)	940 (25.6)	1,290 (29.1)	590 (29.8)	<0.001
Adverse events and physical illnesses [*n*, (%)]^a^					
Sedation	1,799 (17.8)	505 (13.8)	828 (18.7)	466 (23.5)	<0.001
Constipation	1,967 (19.5)	510 (13.9)	949 (21.4)	508 (25.6)	<0.001
Diabetes	795 (7.9)	217 (5.9)	360 (8.1)	218 (11.0)	<0.001
Akathisia	905 (9.0)	296 (8.1)	406 (9.2)	203 (10.2)	0.018
Parkinsonism	1,403 (13.9)	383 (10.4)	677 (15.3)	343 (17.3)	<0.001
Tardive dyskinesia	373 (3.7)	99 (2.7)	166 (3.7)	108 (5.4)	<0.001
Education higher than high school graduate [*n*, (%)]^a^	4,562 (45.2)	1,604 (43.7)	2,032 (45.9)	926 (46.7)	0.076
Regular clinic visits and medication [*n*, (%)]^a^	7,800 (77.4)	3,070 (83.7)	3,387 (76.5)	1,343 (68.1)	<0.001
No period of psychiatric instability in the past year [*n*, (%)]^a^	4,328 (42.9)	1,867 (50.9)	1,865 (42.1)	596 (30.2)	<0.001
No psychiatric hospitalizations in the past year [*n*, (%)]^a^	9,661 (95.8)	3,592 (97.9)	4,252 (96.0)	1,817 (92.0)	<0.001

a Statistical analysis was performed using Pearson’s chi-square test.

b Statistical analysis was performed using the Kruskal–Wallis test.

CGI-S, clinical global impression-severity; SD, standard deviation; SOFAS, social and occupational functioning assessment scale.

### Clinical characteristics by relapse frequency

Overall, 63.6% of the patients (*n* = 6,411) experienced at least one relapse. The clinical characteristics of the total sample and each subgroup (nonrelapsers, low-frequency relapsers, and high-frequency relapsers) are presented in Table 1. No significant differences were observed among the three groups in terms of sex, antidepressant use, presence of akathisia, or education above the high school level. Significant differences were noted for all other factors (all *p* < 0.001, except diagnosis and marital history).

### Factors associated with high social functioning in each group

Univariate logistic regression analyses were performed for all demographic and clinical characteristics. Among them, 26 variables (*p* < 0.001) were identified as candidate factors associated with high social functioning. A multivariate logistic regression analysis was then performed for each subgroup.

**Figure 1.**
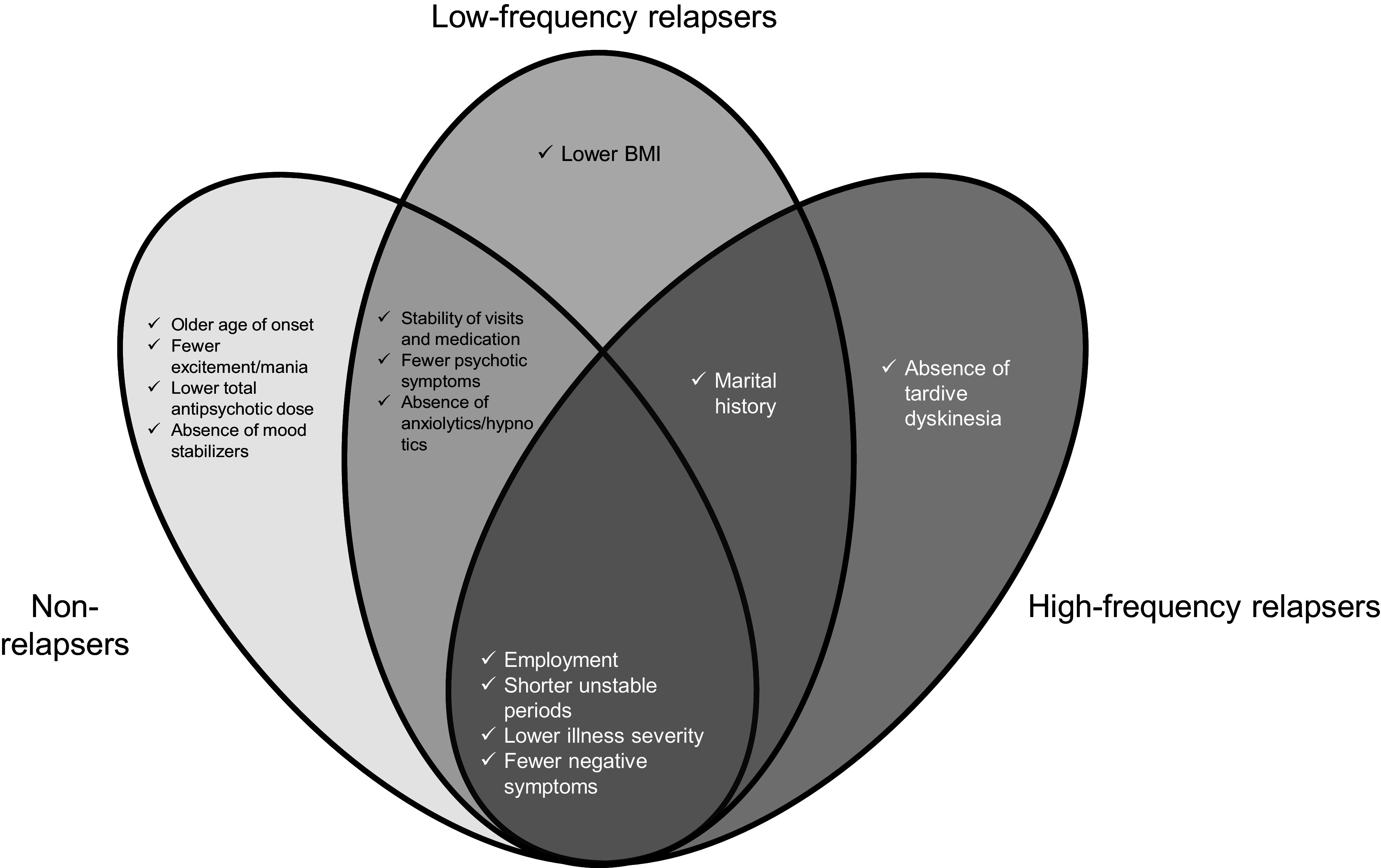
Factors associated with high social functioning by relapse frequency. BMI, body mass index; CGI-S, clinical global impression–severity.

As shown in Table 2 and Figure 1, four factors were consistently associated with high social functioning across all groups: employment, shorter periods of instability during the past year, lower CGI-S scores, and fewer negative symptoms. Factors not common to all groups but associated with high social functioning in specific groups are summarized in Table 3 and Figure 1.

**Table 2.** Factors associated with social functioning across the three groups

	Nonrelapsers			Low-frequency relapsers			High-frequency relapsers		
	Odds ratio	95% CI	*p*-value	Odds ratio	95% CI	*p*-value	Odds ratio	95% CI	*p*-value
Employment	3.636	2.933–4.505	< 0.001	2.825	2.364–3.367	< 0.001	3.215	2.445–4.237	< 0.001
Periods of psychiatric instability during the past year	0.655	0.566–0.758	< 0.001	0.603	0.527–0.689	< 0.001	0.531	0.423–0.665	< 0.001
CGI-S	0.457	0.399–0.524	< 0.001	0.377	0.332–0.428	< 0.001	0.336	0.275–0.410	< 0.001
Negative symptoms	0.541	0.464–0.631	< 0.001	0.619	0.539–0.711	< 0.001	0.602	0.489–0.742	< 0.001

CI, confidence interval; CGI-S, clinical global impression-severity.

**Table 3.** Nonshared social functioning-related factors

	Nonrelapsers			Low-frequency relapsers			High-frequency relapsers		
	Odds ratio	95% CI	*p*-value	Odds ratio	95% CI	*p*-value	Odds ratio	95% CI	*p*-value
Factors shared by non-relapsers and low-frequency relapsers									
Regular clinic visits and medication	1.507	1.171–1.939	0.001	1.511	1.261–1.809	< 0.001			
Psychotic symptoms	0.795	0.683–0.926	0.003	0.786	0.687–0.900	0.001			
Anxiolytics/hypnotics	0.728	0.583–0.91	0.005	0.749	0.617–0.908	0.003			
Factors shared by low-frequency and high-frequency relapsers									
Married				1.414	1.159–1.724	0.001	1.425	1.055–1.919	0.021
Factors unique to each group									
Age of onset	1.013	1.004–1.024	0.005						
Excitement/mania	0.781	0.631–0.967	0.024						
Total anitpysychotic dose (chlorpromazine equivalent)	0.999	0.999–0.999	0.018						
Mood stabilizers	0.645	0.454–0.917	0.014						
Body mass index				0.953	0.915–0.993	0.022			
Tardive dyskinesia							0.473	0.225–0.994	0.048

CI, confidence interval.

In both nonrelapsers and low-frequency relapsers, the stability of visits and medication, fewer psychotic symptoms, and absence of anxiolytic/hypnotic use were associated with high social functioning. Marital history was associated with both low- and high-frequency relapses. None of the factors were shared exclusively by nonrelapsers or high-frequency relapsers.

Among the nonrelapsers, high social functioning was associated with older age at onset, fewer excitement/mania symptoms, lower total antipsychotic dose, and absence of mood stabilizer use. In low-frequency relapsers, high social functioning was associated with a lower BMI. In the high-frequency relapsers, high social functioning was associated with the absence of tardive dyskinesia.

## Discussion

To our knowledge, this is the first nationwide, large-scale, multicenter, cross-sectional survey to investigate outpatient treatment of schizophrenia in Japan. Furthermore, no previous study has examined more than 10,000 cases to explore the differences in factors associated with social functioning according to relapse frequency. After adjusting for other factors through a multivariate logistic regression analysis, four factors – employment, shorter unstable periods during the past year, lower CGI-S scores, and fewer negative symptoms – were consistently associated with high social functioning across all groups. In contrast, group-specific factors associated with social functioning were also identified in nonrelapsers and low-frequency relapsers: stability of visits and medication, fewer psychotic symptoms, and absence of anxiolytic/hypnotic use; in low-frequency and high-frequency relapsers: marital history; in nonrelapsers: older age at onset, fewer excitement/manic symptoms, lower total antipsychotic dose, and absence of mood stabilizer use; in low-frequency relapsers: lower BMI; and in high-frequency relapsers: absence of tardive dyskinesia. These findings suggest that the factors influencing social functioning differ according to the relapse frequency.

The factors common to all groups likely represent the core determinants of social functioning in schizophrenia, regardless of relapse status. Social functioning is a multidimensional construct, and although its definitions vary, it generally encompasses multiple domains (Brissos, Molodynski, Dias, & Figueira, 2011; Mausbach et al., 2009; Peuskens, Gorwood, & Initiative, 2012; Priebe, 2007). Among these, employment is consistently recognized as a central element across assessment scales. The remaining three factors were strongly associated with symptom severity. Previous meta-analyses have shown that the overall symptom severity is significantly associated with social functioning (Handest et al., 2023), with negative symptoms repeatedly identified as the domain most strongly related to social functioning (Escandell et al., 2022; Handest et al., 2023; Strassnig et al., 2018). Our results are consistent with previous evidence and confirm similar trends in Japanese outpatients with schizophrenia.

Group-specific analyses provided additional insights. Three domains emerged among nonrelapsers and low-frequency relapsers: age at onset, positive symptom clusters, and psychotropic medication-related factors. A later age of onset has been associated with better social functioning (Immonen, Jaaskelainen, Korpela, & Miettunen, 2017), possibly due to the preserved time for social skill development and fewer neurodevelopmental abnormalities seen in early onset cases (Guo et al., 2022; Linke et al., 2015), and relatively milder cognitive impairment compared to early-onset schizophrenia (Rajji, Ismail, & Mulsant, 2009). In our study, this association was limited to nonrelapsers, possibly reflecting the progressive impact of negative symptoms and cognitive impairment in patients with repeated relapses.

Medication-related factors also appeared to be relevant. The use of anxiolytics/hypnotics, higher total antipsychotic doses, and mood stabilizers was associated with lower social functioning. Benzodiazepines, commonly prescribed as anxiolytics or hypnotics, are known to impair cognition and cause sedation (Dold et al., 2012; Pottie et al., 2018; Stewart, 2005), whereas high-dose antipsychotics may also lead to cognitive decline and excessive sedation (Yoshida & Takeuchi, 2021). Despite limited evidence regarding cognition or functioning in schizophrenia, mood stabilizers, particularly non-lithium agents, may exert sedative effects (Bai et al., 2019). These findings suggest that medication use and dosing affect social functioning via cognition and arousal. Notably, such associations were restricted to patients with a low relapse frequency. In high-frequency relapsers, sedative effects may aid in symptom control, thereby offsetting potential functional decline.

Psychotic symptoms, excitement/mania, and stability of visits and medications were also identified as important factors. Positive symptoms have been associated with social functioning in previous studies, although their impact is less robust than that of negative symptoms (Escandell et al., 2022; Handest et al., 2023). In patients with frequent relapses, functional impairment may be driven by persistent cognitive decline, whereas overt positive symptoms may exert stronger effects in those with fewer relapses. The stability of visits and medications is directly associated with “independent living,” a core domain of social functioning, and its central component – medication adherence – is particularly linked to relapse and symptom exacerbation (Olivares, Sermon, Hemels, & Schreiner, 2013). As psychotic symptoms and excitement/mania are frequently observed as prominent features during relapse or symptom worsening, these symptoms, together with the stability of visits and medications, may have been identified as factors associated with social functioning, specifically in nonrelapsers and low-frequency relapsers.

Finally, marital history and absence of tardive dyskinesia were linked to higher social functioning in relapsers. Spousal support, including medication management, clinic attendance, and maintenance of daily routines, may facilitate functional recovery, which is consistent with the findings of previous meta-analyses (Pharoah, Mari, Rathbone, & Wong, 2010). Tardive dyskinesia, often associated with long-term high-dose antipsychotics (Solmi, Pigato, Kane, & Correll, 2018), may impair functioning through motor symptoms and stigma, although one study has disputed its direct impact (Nadesalingam et al., 2022). In this study, its relevance was confined to high-frequency relapsers, potentially reflecting the cumulative illness burden, brain volume loss (Haijma et al., 2013), and cognitive decline (Kubota et al., 2015), which may have amplified its impact on daily functioning. The availability of treatments, such as vesicular monoamine transporter 2 inhibitors (Solmi et al., 2025), highlights opportunities to mitigate their effects and improve outcomes in these patients.

### Limitations

This study had several limitations. First, owing to its cross-sectional design, causal relationships could not be inferred, and it remains unclear whether the identified factors directly contribute to social functioning. However, the robustness of the findings is supported by the large sample size of over 10,000 patients and the adjustment for potential confounders through a multivariate logistic regression analysis. Second, relapse frequency was evaluated based on medical records and clinician judgment, introducing possible heterogeneity in the criteria used. Third, social functioning was assessed solely using the SOFAS, which may not sufficiently reflect patients’ or families’ subjective perspectives or objective indicators such as employment duration or quality of interpersonal relationships. Fourth, this study did not confirm whether patients met the criteria for treatment-resistant schizophrenia (TRS). Although only one patient in this study was prescribed clozapine, this does not necessarily imply that the majority of the patients did not have TRS, given that clozapine is notably underutilized in Japan (Bachmann et al., 2017). As patients with TRS typically experience more frequent hospitalizations and relapses (Correll, Brevig, & Brain, 2019), the clinical characteristics of TRS are likely captured primarily within the high-frequency relapse group; therefore, the lack of formal TRS identification might have had only a limited impact on our overall findings. Fifth, because the data on relapse status were collected as categorical variables, we were unable to analyze the number of relapses as a continuous variable to investigate its specific impact on social functioning. Sixth, although clinical assessments in this study were performed by physicians who were in charge of the patients and were well-trained, the qualitative assessment of the evaluations themselves was not conducted. Therefore, the possibility of inter-rater or inter-facility variability cannot be excluded. Finally, because the study was limited to outpatients, the findings may not be generalizable to severely ill inpatients. Nonetheless, given that most patients with schizophrenia are treated in an outpatient setting, this study provides valuable insights into real-world clinical practice.

In conclusion, this large-scale cross-sectional study of outpatients with schizophrenia across Japan demonstrated that factors associated with social functioning differ according to relapse frequency. Across all groups, employment, shorter periods of instability in the past year, lower clinical severity, and fewer negative symptoms were consistently associated with improved social functioning. In nonrelapsers and low-frequency relapsers, additional attention should be paid to manifested symptoms, such as psychotic symptoms and excitement/mania, as well as the impact of pharmacological treatment. In contrast, in high-frequency relapsers, the absence of tardive dyskinesia was associated with better social functioning. These findings suggest the need to stratify intervention strategies according to relapse frequency, with an integrated consideration of symptoms, treatment, and side-effect management. Future longitudinal studies are warranted to clarify causal relationships and incorporate neurocognitive and neurobiological factors into a more comprehensive understanding.

## Supporting information

10.1017/S0033291726104231.sm001 Takekita et al. supplementary material Takekita et al. supplementary material

## Data Availability

The data that support the findings of this study are not publicly available due to ethical and legal restrictions related to patient privacy and the terms of institutional approvals. In accordance with these restrictions, the dataset is available only to the project investigators named on the ethics applications and cannot be shared with researchers outside the project team.

## Supplementary material

The supplementary material for this article can be found at http://doi.org/10.1017/S0033291726104231.
