# Supplementary material for: Factors associated with social functioning by relapse frequency in Japanese outpatients with schizophrenia: the Multicenter Treatment Survey and Assessments for Schizophrenia in Psychiatric Clinics (MUSASI)
Source: Psychol Med. 2026 May 29;56:e159. doi: 10.1017/S0033291726104231 (PMC13234521; doi:10.1017/S0033291726104231)
Supplement: Takekita et al. supplementary material [file S0033291726104231sup001.docx]

Supplementary Tables S1. STROBE Statement—checklist of items that should be included in reports of observational studies

|  | Item No. | Recommendation | Page  No. | Relevant text from manuscript |
| --- | --- | --- | --- | --- |
| **Title and abstract** | 1 | 1. Indicate the study’s design with a commonly used term in the title or the abstract | 4 | This nationwide cross-sectional study, the... schizophrenia-related disorders were analyzed. |
|  |  | 1. Provide in the abstract an informative and balanced summary of what was done and what was found | 4-5 | Impaired social functioning... stratified intervention strategies. |
| Introduction | | | |  |
| Background/rationale | 2 | Explain the scientific background and rationale for the investigation being reported | 6-7 | Schizophrenia is one of the most... support and developing treatment strategies. |
| Objectives | 3 | State specific objectives, including any prespecified hypotheses | 7 | Therefore, this study aimed to investigate... outpatients with schizophrenia. |
| Methods | | | |  |
| Study design | 4 | Present key elements of study design early in the paper | 8 | The Multicenter Treatment Survey... in psychiatric clinics across Japan. |
| Setting | 5 | Describe the setting, locations, and relevant dates, including periods of recruitment, exposure, follow-up, and data collection | 8 | The Multicenter Treatment Survey and... in psychiatric clinics across Japan.  Most psychiatrists who participated... the Ministry of Health, Labour and Welfare of Japan. |
| Participants | 6 | (*a*) *Cohort study*—Give the eligibility criteria, and the sources and methods of selection of participants. Describe methods of follow-up  *Case-control study*—Give the eligibility criteria, and the sources and methods of case ascertainment and control selection. Give the rationale for the choice of cases and controls  *Cross-sectional study*—Give the eligibility criteria, and the sources and methods of selection of participants | 8 | Participants were patients diagnosed with... receiving treatment at the clinics.  Psychiatrists were asked to... the first patient seen and completing the questionnaire. |
|  |  | (*b*) *Cohort study*—For matched studies, give matching criteria and number of exposed and unexposed  *Case-control study*—For matched studies, give matching criteria and the number of controls per case | N/A | Not applicable |
| Variables | 7 | Clearly define all outcomes, exposures, predictors, potential confounders, and effect modifiers. Give diagnostic criteria, if applicable | 8-9 | Diagnosis: Participants were patients diagnosed... treatment at the clinics.  Variables: The questionnaire included questions... pharmacological treatment, and side effects.  Outcome: Following the classification of... social functioning” groups. |
| Data sources/ measurement | 8* | For each variable of interest, give sources of data and details of methods of assessment (measurement). Describe comparability of assessment methods if there is more than one group | 8-9 | Psychiatrists were asked to retrospectively review the medical records... functioning in four categories: 0:1–40, 1:41–60, 2:61–80, and 3:81–100. |
| Bias | 9 | Describe any efforts to address potential sources of bias | 8, 10 | Selection/Information Bias: Psychiatrists were asked to retrospectively... completing the questionnaire.  Confounding Bias: Variables with p < 0.001 were subsequently included in ... to relapse frequency (non-relapsers, low-frequency relapsers, and high-frequency relapsers). |
| Study size | 10 | Explain how the study size was arrived at | 12 | Of the 1,544 facilities invited to... leaving 10,081 patients for the analysis. |

Continued on next page

| Quantitative variables | 11 | Explain how quantitative variables were handled in the analyses. If applicable, describe which groupings were chosen and why | 9-10 | Relapse: Relapse frequency was categorized... relapsers (≥3 relapses).  SOFAS: The modified SOFAS rated... 3:81–100.  Cutoff: Following the classification of... relapse frequency groups. |
| --- | --- | --- | --- | --- |
| Statistical methods | 12 | (*a*) Describe all statistical methods, including those used to control for confounding | 10 | Univariate logistic regression analyses... to relapse frequency (non-relapsers, low-frequency relapsers, and high-frequency relapsers). |
|  |  | (*b*) Describe any methods used to examine subgroups and interactions | 10, 13 | Variables with p < 0.001 were ... to relapse frequency (non-relapsers, low-frequency relapsers, and high-frequency relapsers).  A multivariate logistic regression analysis was then performed for each subgroup. |
|  |  | (*c*) Explain how missing data were addressed | 12 | Data were obtained from... patients for the analysis. |
|  |  | (*d*) *Cohort study*—If applicable, explain how loss to follow-up was addressed  *Case-control study*—If applicable, explain how matching of cases and controls was addressed  *Cross-sectional study*—If applicable, describe analytical methods taking account of sampling strategy | 10 | Cross-sectional study: To compare demographic and... *chi-square tests were used.* |
|  |  | (*e*) Describe any sensitivity analyses | N/A | Not applicable |
| Results | | | | |
| Participants | 13* | 1. Report numbers of individuals at each stage of study—eg numbers potentially eligible, examined for eligibility, confirmed eligible, included in the study, completing follow-up, and analysed | 12 | Of the 1,544 facilities invited to... leaving 10,081 patients for the analysis. |
|  |  | 1. Give reasons for non-participation at each stage | 12 | Data were obtained from... patients for the analysis. |
|  |  | 1. Consider use of a flow diagram | N/A | Not performed. |
| Descriptive data | 14* | 1. Give characteristics of study participants (eg demographic, clinical, social) and information on exposures and potential confounders | 12 | Demographic characteristics of participants... 55.8% (n = 5,631).  The clinical characteristics... are presented in Table 1. |
|  |  | 1. Indicate number of participants with missing data for each variable of interest | 12-13 | The clinical characteristics of... presented in Table 1.  Significant differences were noted... except diagnosis marital history). |
|  |  | 1. *Cohort study*—Summarise follow-up time (eg, average and total amount) | N/A | Not applicable. |
| Outcome data | 15* | *Cohort study*—Report numbers of outcome events or summary measures over time | N/A | Not applicable. |
|  |  | *Case-control study—*Report numbers in each exposure category, or summary measures of exposure | N/A | Not applicable. |
|  |  | *Cross-sectional study—*Report numbers of outcome events or summary measures | 12 | The proportion of patients... 55.8% (n = 5,631*).* |
| Main results | 16 | 1. Give unadjusted estimates and, if applicable, confounder-adjusted estimates and their precision (eg, 95% confidence interval). Make clear which confounders were adjusted for and why they were included | 13 | Adjusted estimates**:** As shown in Table 2... and fewer negative symptoms.  Unadjusted estimates**:** Univariate logistic regression analyses were performed for all demographic and clinical characteristics. |
|  |  | 1. Report category boundaries when continuous variables were categorized | 9 | Following the classification of functional remission ... and “low social functioning” groups. |
|  |  | 1. If relevant, consider translating estimates of relative risk into absolute risk for a meaningful time period | N/A | Not performed / Not applicable. This cross-sectional study reports Odds Ratios (ORs), and absolute risk was not calculated or reported. |

Continued on next page

| Other analyses | 17 | Report other analyses done—eg analyses of subgroups and interactions, and sensitivity analyses | 10, 13-14 | Subgroups: Variables with p < 0.001 were ... (non-relapsers, low-frequency relapsers, and high-frequency relapsers).  Group-specific factors: In both non-relapsers and low-frequency relapsers... with the absence of tardive dyskinesia. |
| --- | --- | --- | --- | --- |
| Discussion | | | | |
| Key results | 18 | Summarise key results with reference to study objectives | 15 | After adjusting for other... in high-frequency relapsers: absence of tardive dyskinesia. |
| Limitations | 19 | Discuss limitations of the study, taking into account sources of potential bias or imprecision. Discuss both direction and magnitude of any potential bias | 19 | This study had several... valuable insights into real-world clinical practice. |
| Interpretation | 20 | Give a cautious overall interpretation of results considering objectives, limitations, multiplicity of analyses, results from similar studies, and other relevant evidence | 19-20 | In conclusion, this large-scale cross-sectional... consideration of symptoms, treatment, and side-effect management. |
| Generalisability | 21 | Discuss the generalisability (external validity) of the study results | 19 | Finally, because the study... insights into real-world clinical practice. |
| Other information | |  | | |
| Funding | 22 | Give the source of funding and the role of the funders for the present study and, if applicable, for the original study on which the present article is based | 22 | YT has received grant... Novartis Pharma, and Otsuka. |

*Give information separately for cases and controls in case-control studies and, if applicable, for exposed and unexposed groups in cohort and cross-sectional studies.

**Note:** An Explanation and Elaboration article discusses each checklist item and gives methodological background and published examples of transparent reporting. The STROBE checklist is best used in conjunction with this article (freely available on the Web sites of PLoS Medicine at http://www.plosmedicine.org/, Annals of Internal Medicine at http://www.annals.org/, and Epidemiology at http://www.epidem.com/). Information on the STROBE Initiative is available at www.strobe-statement.org.
